# Are Everolimus-Eluting Stents Associated With Better Clinical Outcomes Compared to Other Drug-Eluting Stents in Patients With Type 2 Diabetes Mellitus? A Systematic Review and Meta-Analysis

**DOI:** 10.1097/MD.0000000000003276

**Published:** 2016-04-08

**Authors:** Pravesh Kumar Bundhun, Manish Pursun, Abhishek Rishikesh Teeluck, Man-Yun Long

**Affiliations:** From the Institute of Cardiovascular Diseases (PKB, M-YL), the First Affiliated Hospital of Guangxi Medical University, Nanning, Guangxi, China, and First Affiliated Hospital of Guangxi Medical University (MP, ART), Nanning, Guangxi, China.

## Abstract

Controversies still exist with the use of Everolimus-Eluting Stents (EES) compared to other Drug-Eluting Stents (DES) in patients with Type 2 Diabetes Mellitus (T2DM). Therefore, in order to solve this issue, we aim to compare the 1-year adverse clinical outcomes between EES and non-EE DES with a larger number of patients with T2DM.

Medline, EMBASE, PubMed databases, as well as the Cochrane library were searched for randomized controlled trials (RCTs) and observational studies (OS) comparing EES and non-EE DES in patients with T2DM. One-year adverse outcomes were considered as the clinical endpoints in this study. Odd ratios (OR) with 95% confidence interval (CI) were used to express the pooled effect on discontinuous variables and the pooled analyses were performed with RevMan 5.3.

Ten studies consisting of a total of 11,981 patients with T2DM (6800 patients in the EES group and 5181 in the non-EE DES group) were included in this meta-analysis. EES were associated with a significantly lower major adverse cardiac events (MACEs) with OR: 0.83, 95% CI: 0.70–0.98, *P* = 0.03. Revascularization including target vessel revascularization (TVR) and target lesion revascularization (TLR) were also significantly lower in the EES group with OR: 0.62, 95% CI: 0.40–0.94, *P* = 0.03 and OR: 0.74, 95% CI: 0.57–0.95, *P* = 0.02, respectively. Also, a significantly lower rate of stent thrombosis with OR: 0.63, 95% CI: 0.46–0.86, *P* = 0.003 was observed in the EES group. However, a similar mortality rate was reported between the EES and non-EE DES groups.

During this 1-year follow-up period, EES were associated with significantly better clinical outcomes compared to non-EE DES in patients suffering from T2DM. However, further research comparing EES with non-EE DES in insulin-treated and noninsulin-treated patients with T2DM are recommended.

## INTRODUCTION

Adverse cardiovascular outcomes have significantly decreased with the introduction of drug-eluting stents (DES). Repeated revascularization has also reduced in patients undergoing percutaneous coronary intervention (PCI) with DES.^1^ These are the possible reasons why first-generation DES such as paclitaxel-eluting stents (PES) and sirolimus-eluting stents (SES) were preferred to Bare Metal Stents (BMS).^2,3^ Nowadays, many second-generation DES have also been approved for use in PCI centers, among which everolimus-eluting stents (EES) have shown to be associated with favorable clinical outcomes in patients suffering from coronary artery diseases (CAD).^4^ However, whether those favorable outcomes apply to different subgroups such as in patients with Type 2 Diabetes Mellitus (T2DM) is still controversial.

Generally, patients with T2DM have worse clinical outcomes after PCI.^5^ The rate of repeated revascularization in patients with T2DM is also significantly higher compared to patients without T2DM. Several studies have shown the head-to-head comparison between EES and other DES in patients with T2DM. For example, the study by Park et al showed that EES were considered equally effective when compared to zotarolimus-eluting stents (ZES) in patients with T2DM at 1 year after PCI.^6^ Moreover, another study by Stone et al, comparing EES with PES in patients with T2DM, did not show any benefit associated with EES during a follow-up of 2 years.^7^

However, the study by Muramatsu et al comparing EES with bioresorbable vascular scaffold (BVS) in patients with T2DM showed a lower incidence of target lesion failure, cardiac death, target lesion revascularization (TLR), and myocardial infarction (MI) at 1 year after PCI-associated with EES.^8^ Also, the recently published study by Kaul comparing EES with PES in patients with T2DM showed the association of a significantly lower rate of stent thrombosis (ST) with EES.^9^

As different results were reported when EES were compared with different types of DES, at times favoring EES whereas sometimes favoring the other DES, and because a limited number of T2DM patients were analyzed in previously published studies, we aim to compare the 1-year adverse clinical outcomes between EES and non-EE DES using a larger number of patients with T2DM, in order to assess whether EES are associated with better or similar adverse clinical outcomes compared to other DES.

## METHOD

### Data Sources and Search Strategy

Medline, EMBASE, PubMed databases, as well as the Cochrane library were searched for randomized controlled trials (RCTs) and observational studies (OS) by typing the words or phrases “everolimus-eluting stents and diabetes mellitus” and “drug-eluting stents and diabetes mellitus.” Abbreviations such as “EES, DES, PCI, T2DM, DM” have also been used during the search process. To further enhance this search, the phrase “first-generation DES and second-generation DES” were also used. No language restriction was applied.

### Inclusion and Exclusion Criteria

Studies were included if:

1. They were RCTs or OS comparing EES with non-EE DES.
2. The comparison involved patients with T2DM.
3. They reported adverse clinical outcomes as their endpoints.
4. They had a follow-up period of 1 year.

Studies were excluded if:

1. They were neither RCTs nor OS.
2. The comparison did not involve patients with T2DM.
3. EES were compared with BMS instead of other DES.
4. Adverse clinical outcomes were not reported among their endpoints.
5. They had a shorter follow-up periods of several months.

### Defining Terms, Outcomes, and Follow-Up

Non-EE DES: were defined as any type of DES excluding EES: for example, PES, SES, ZES, and BVS.

Adverse clinical outcomes analyzed in the present study included:

- Major Adverse Cardiac Events (MACEs)—MACEs which were also referred to as the composite endpoints, included death, fatal or nonfatal MI, stroke or repeated target vessel revascularization (TVR); or any other revascularization.
- All-cause mortality (cardiac and noncardiac deaths).
- MI which could be fatal, nonfatal MI, spontaneous MI, Q wave and non Q wave MI.
- Revascularization (TVR and TLR).
- Stent Thrombosis (ST)—ST which was defined according to the Academic Research Consortium (ARC) included definite, probable or possible ST. However, if a clear definition of ST was not provided, ST was assumed to have been defined by ARC and these data have been included in our meta-analysis.

Most of the included studies had a follow-up period of 1 year. Table 1 shows the reported outcomes and follow-up periods of the included studies.

**TABLE 1:**
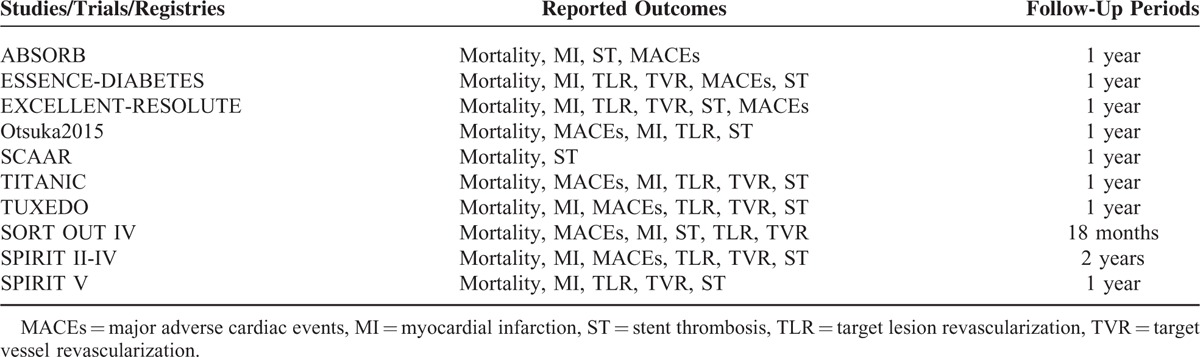
Reported Outcomes and Follow-Up Periods

### Data Extraction and Quality Assessment

Three authors (PKB, MP, and ART) independently assessed whether the studies were eligible or not, and then reviewed the data. Information regarding the study type, the year of publication, the total number of patients with T2DM, the patient characteristics, types of DES, the number of patients associated with EES and non-EE DES, and the adverse clinical outcomes reported as well as the follow-up periods was systematically extracted. If one of the authors could not reach a decision or disagreed about including certain studies, disagreements were discussed among the authors, and if the authors could not reach a consensus, disagreements were resolved by another author (MYL). The bias risk of trials was assessed with the components recommended by the Cochrane Collaboration.^10^

### Methodological Quality and Statistical Analysis

Recommendations of the Preferred Reporting Items for Systematic Reviews and Meta-Analyses (PRISMA) statement ^11^ were considered in this study. Heterogeneity across trials were assessed using the Cochrane Q-statistic (*P* ≤ 0·05 was considered significant whereas *P* > 0.05 was considered as statistically insignificant) and *I*^2^-statistic. *I*^2^ described the percentage of total variation across studies; that is, whether the variation was due to heterogeneity rather than chance. A value of 0% indicated no heterogeneity, and increasing values indicated increasing heterogeneity. If *I*^2^ was >50%, a random effect model was used. However, if *I*^2^ was <50%, a fixed effect model used. Publication bias was visually estimated by assessing funnel plots. We calculated odd ratios (OR) and 95% confidence intervals (CIs) for categorical variables. The pooled analyses were performed with RevMan 5.3 software.

Ethics: Ethical approval was not necessary as this study is a systematic review and meta-analysis.

## RESULTS

### Study Selection

**FIGURE 1:**
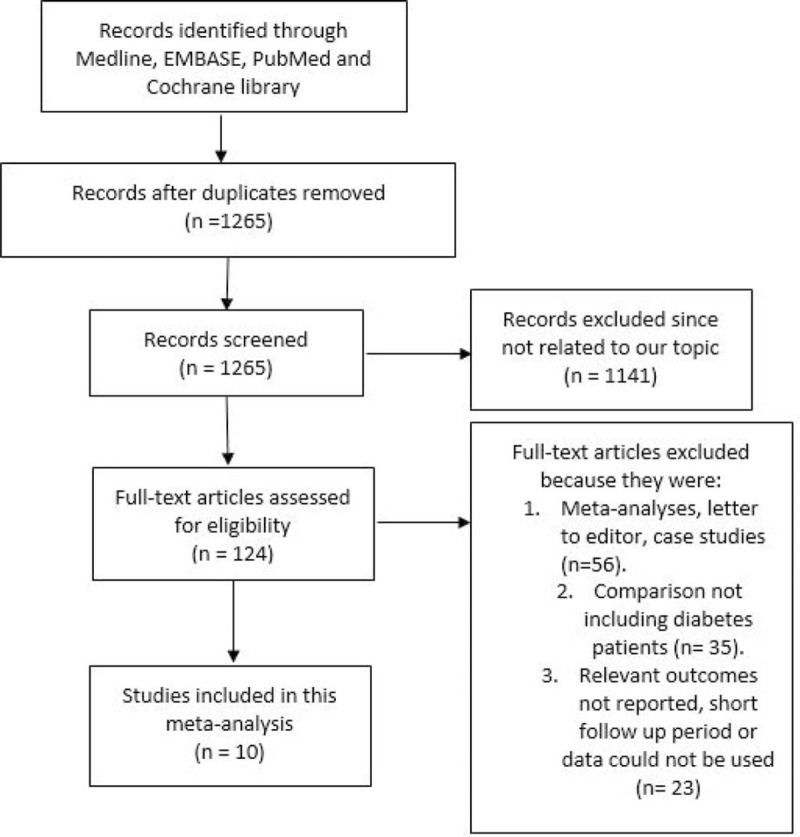
The flow diagram for the study selection.

A number of 1456 articles have been identified from Medline, EMBASE, PubMed databases, as well as from the Cochrane library. After eliminating the duplicate studies and studies not related to our topic, 124 full text articles were finally assessed for eligibility. Among those 124 studies, 56 articles were further eliminated because they were meta-analyses, letter to editors or case studies. Moreover, a further 35 studies were eliminated because they did not compare the clinical outcomes between EES and non-EE DES in patients with T2DM, but instead, compared EES with non-EE DES in the general population who underwent PCI. Another 23 studies were eliminated for the following reasons: they did not report the corresponding clinical endpoints, they had a shorter follow-up period, or their data could not be used. Finally, 10 studies were selected and included in this meta-analysis.^9,12–19^ The flow diagram for the study selection has been illustrated in Figure 1.

A total of 11,981 patients with T2DM including 6800 patients in the EES group and 5181 patients in the non-EE DES group were analyzed in this study. Table 2 represents the general features of the included studies.

**TABLE 2:**
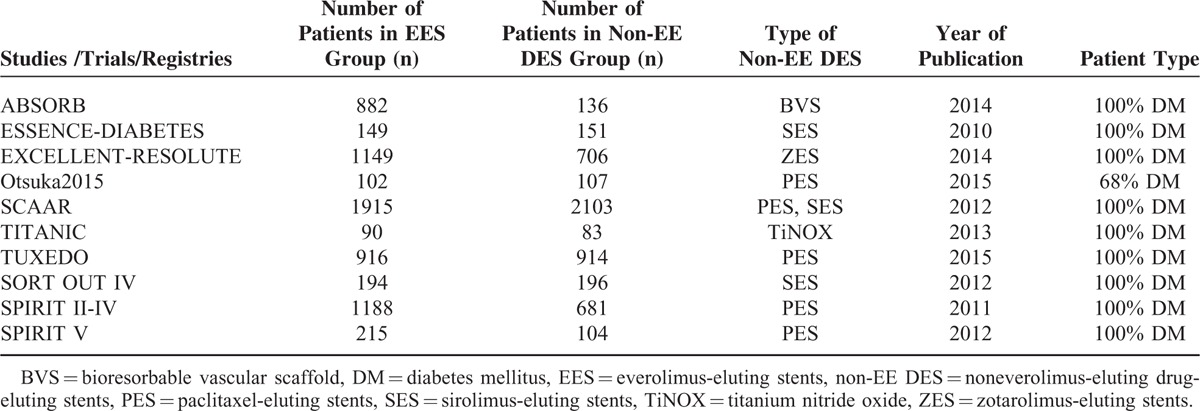
General Features of the Included Studies

### Baseline Features

Table 3 represents the baseline characteristics of the included studies. Patient age was almost similar in the experimental and the control groups. SCAAR registry had the highest number of patients with insulin-treated diabetes mellitus (ITDM) followed by the TUXEDO study. The percentage of male patients was higher compared to female patients. SCAAR registry had the highest number of smokers in both categories of patients. The percentages of patients suffering from hypertension and dyslipidemia were high in majority of the studies. Overall, there were no significant differences in the baseline features among patients from the EES and non-EE DES groups.

**TABLE 3:**
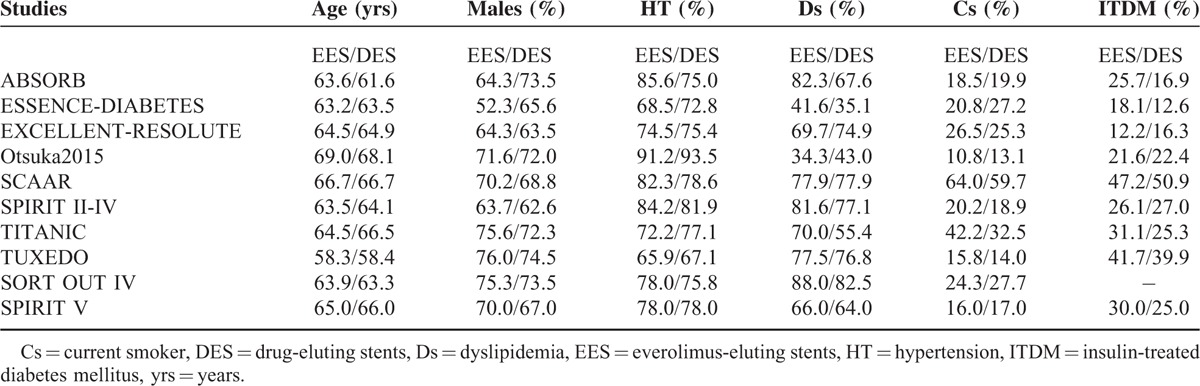
Baseline Characteristics of the Included Studies

### Analyzed Data

**FIGURE 2:**
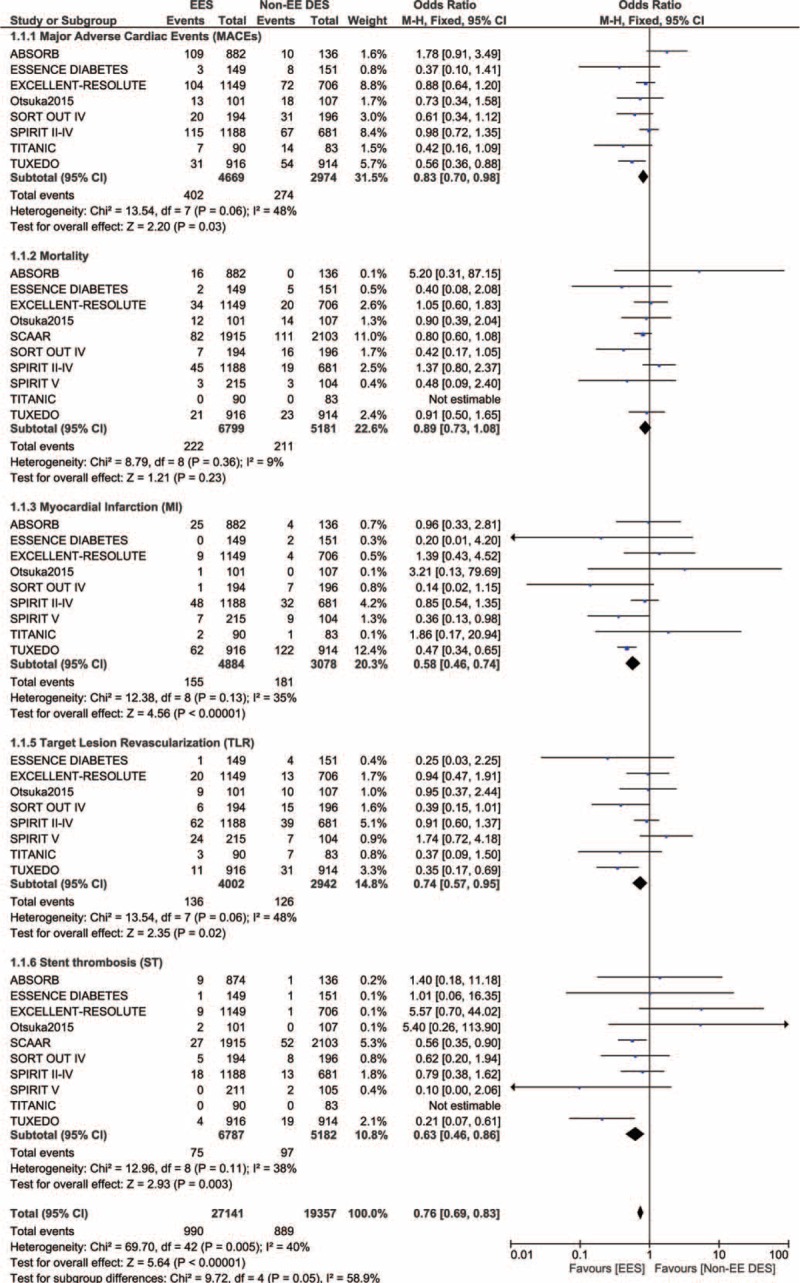
Forest plot showing the adverse clinical outcomes between EES and Non-EE DES in patients with T2DM. EES = everolimus-eluting stents, non-EE DES = noneverolimus-eluting drug-eluting stents, T2DM = type 2 diabetes mellitus.

Table 4 represents the results of this meta-analysis. A total of 4669 patients from the EES group and 2974 patients from the non-EE DES group were analyzed by a fixed effect model. EES were associated with a significantly lower rate of MACEs with OR: 0.83, 95% CI: 0.70–0.98, *P* = 0.03 compared to non-EE DES. Among the 6799 patients in the EES group and 5181 patients in the non-EE DES group analyzed for all cause death, a similar mortality rate has been reported with OR: 0.89, 95% CI: 0.73–1.08, *P* = 0.23. MI which was considered as one of the components of MACEs, was also significantly lower in the EES group with OR: 0.58, 95% CI: 0.46–0.74, *P* < 0.00001. TLR was also significantly lower in the EES group with OR: 0.74, 95% CI: 0.57–0.95, *P* = 0.02. ST which was defined according to the ARC, was also significantly lower in the EES group with OR: 0.63, 95% CI: 0.46–0.86, *P* = 0.003. These results have been illustrated in Figure 2.

**TABLE 4:**
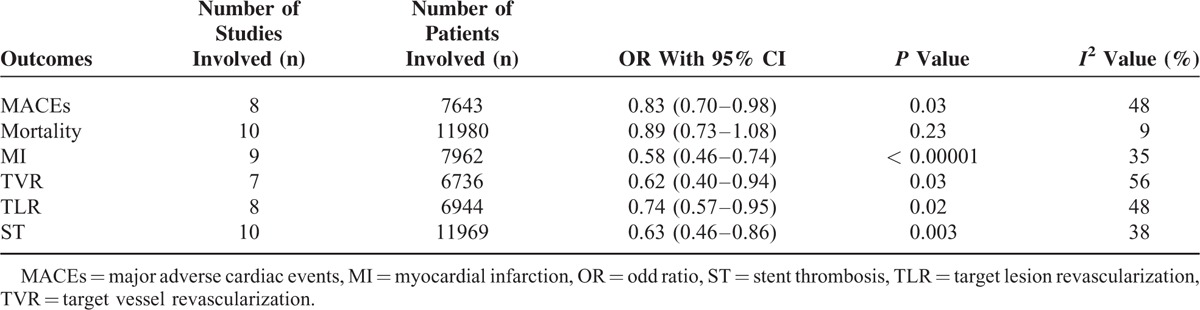
Results of this Meta-Analysis

**FIGURE 3:**
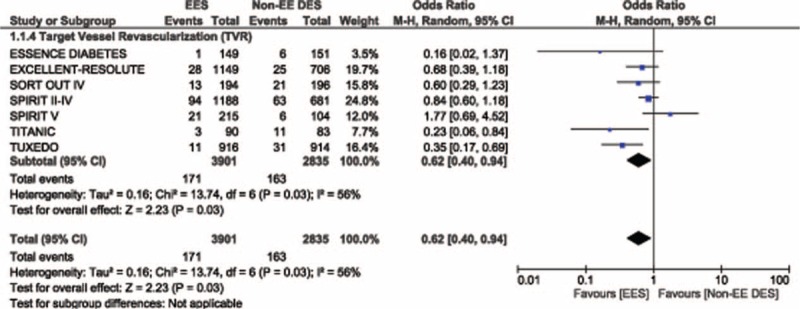
Forest plot illustrating the result for target vessel revascularization between EES and non-EE DES in patients with T2DM. EES = everolimus-eluting stents, non-EE DES = noneverolimus-eluting drug-eluting stents, T2DM = type 2 diabetes mellitus.

Because heterogeneity was higher when comparing TVR between these 2 DES groups, a random effect model was used for the analysis of this outcome. Among 6736 patients analyzed, TVR was also significantly lower in the EES group with OR: 0.62, 95% CI: 0.40–0.94, *P* = 0.03. This result has been illustrated in Figure 3.

**FIGURE 4:**
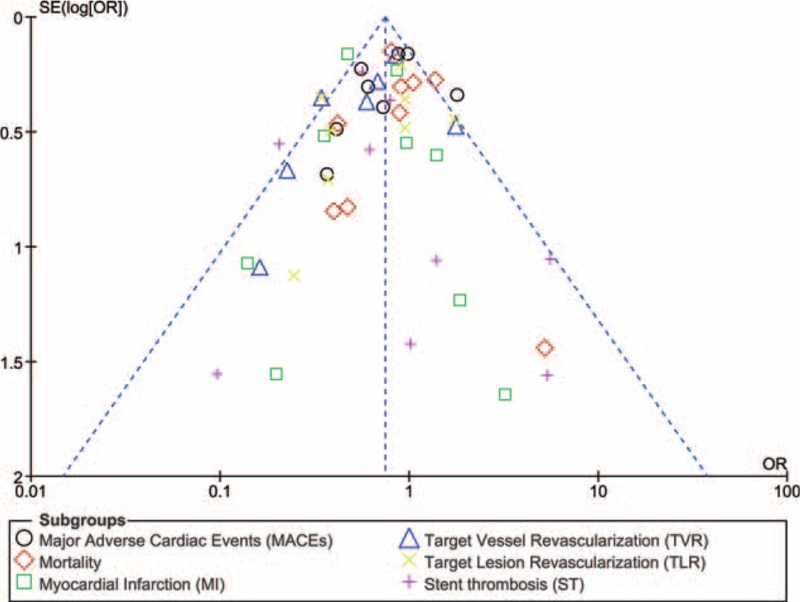
Funnel plot showing the sensitivity analysis.

For all of the above analyses, sensitivity analyses yielded consistent results. Based on a visual inspection of the funnel plot analyzing the adverse clinical outcomes, there has been almost no evidence of publication bias for the included studies. The funnel plot has been illustrated in Figure 4.

## DISCUSSION

Our results showed that EES were associated with a significantly lower rate of MACEs, revascularization and ST compared to the non-EE DES. However, there was no significant difference in mortality between these 2 groups. Possible reasons explaining the better clinical outcomes associated with EES, including a lower rate of ST, have previously been discussed.^20–23^

Similar to our study, another meta-analysis including 13 RCTs involving >30% of patients with T2DM, and comparing the outcomes between EES and non-EE DES in the general population with CAD showed a significantly reduced ST, TVR, and MI associated with EES compared to the non-EE DES group.^4^ The study published by Kaul et al also supports our results.^9^ His study which compared EES with PES in patients with T2DM showed PES to be associated with a significantly higher rate of MI (32.2% vs 1.2%), revascularization (3.4% vs 1.2%), and ST (2.1% vs 0.4%) compared to EES. The study published by Lopez Minguez also showed EES to be superior to titanium DES in terms of both clinical and angiographic endpoints in patients with T2DM.^17^ Moreover, the meta-analysis published by Palmerini et al comparing ST in patients treated with EES and other DES also showed a significantly lower rate of ST associated with EES during a follow-up period of at least 2 years.^24^

However, many other studies also showed results which were completely different from our result. For example, Stone et al investigated the differential clinical responses to EES and PES in patients with and without T2DM and concluded that EES were more effective and safe in nondiabetics; however, no benefit was observed between these 2 DES in patients with T2DM. The pilot study involving the Naples-Diabetes trial suggested that EES were associated with a higher rate of MACEs during a 3-year follow-up period compared to PES and SES in patients with T2DM.^25^ However, his study included patients with T2DM who had major complications such as diabetic retinopathy or nephropathy and poor metabolic control. Additionally, the study by Kereiakes comparing the outcomes in patients with T2DM and non-T2DM treated with EES or PES showed similar clinical outcomes between EES and PES during a follow-up period of 1 year.^26^

This study is new in the way that it is the first study comparing EES and non-EE DES with a larger number of T2DM patients. All the other studies mentioned previously included only a small population of patients with T2DM. Moreover, our study did not include data from unpublished studies. Finally, in contrast to other studies, this study did not include participants from only 1 region, as reported in the study by Kaul et al.^9^ Our study included patients from different parts of the globe and obtaining results that can be applied universally.

## LIMITATIONS

This study has several limitations. First of all, due to the small population size of patients with T2DM, the result of this analysis could be affected to an extent. Moreover, we have included a study which did not consist of 100% patients with T2DM. However, as >60% of the patients had T2DM, and because this study consisted of a very small number of patients compared to the other included studies, and considering the fact that the inclusion or exclusion of this study from our meta-analysis will not have a great impact on our result, we have included it in this current meta-analysis. In addition, 2 studies, 1 with a follow-up period of 18 months and the other one with a follow-up period of 2 years, were also included in this analysis.

## CONCLUSION

During this 1-year follow-up period, EES were associated with significantly better clinical outcomes compared to the non-EE DES in patients with T2DM. However, further researches comparing EES with non-EE DES in patients with ITDM and NITDM are recommended.
